# A Preoperative Risk Prediction Model for Lymph Node Examination of Stage I-III Colon Cancer Patients: A Population-Based Study

**DOI:** 10.7150/jca.41056

**Published:** 2020-03-05

**Authors:** Yuliuming Wang, Xu Guan, Yukun Zhang, Zhixun Zhao, Zhifeng Gao, Haipeng Chen, Weiyuan Zhang, Zheng Liu, Zheng Jiang, Yinggang Chen, Guiyu Wang, Xishan Wang

**Affiliations:** 1 Department of Colorectal Surgery, the Second Affiliated Hospital of Harbin Medical University, Harbin, China.; 2 Department of Colorectal Surgery, National Cancer Center / National Clinical Research Center for Cancer / Cancer Hospital, Chinese Academy of Medical Sciences and Peking Union Medical College, Beijing, China.

**Keywords:** prediction model, colon cancer, lymph node examination

## Abstract

**Background**: Lymph node examination is a prognostic indicator for colon cancer (CC) patients. The aim of this study was to develop and validate a preoperative risk prediction model for inadequate lymph node examination.

**Methods**: 24284 patients diagnosed as stage I-III CC between 2010-2014 were extracted from SEER database and randomly divided into development cohort (N=12142) and internal validation cohort (N=12142). 680 patients diagnosed as stage I-III CC between 2012-2014 were extracted from our hospital as external validation cohort. Logistic regression analysis was performed and risk score of each factor was calculated according to model formula. Model discrimination was assessed using C-statistics.

**Results**: Preoperative risk factors were identified as gender, age, tumor site and tumor size. Patients with total risk score of 0-6 were considered as low risk group while patients scored ≥13 were considered as high risk group. The model had good discrimination and calibration in all cohorts and could apply to patients in the SEER database (American population) and patients in our hospital (Chinese population).

**Conclusions**: The model could accurately predict the risk of inadequate lymph node examination before surgery and might provide useful reference for surgeons and pathologists.

## Introduction

Colorectal cancer (CRC) is one of the most common cancers in the world 1. In China, CRC is the third and fourth most common cancer in urban and rural districts, respectively. Lymph node examination has been used as a prognostic indicator associated with survival, as well as one key factor that guides adjuvant chemotherapy after radical resection of CRC 2-3. In addition, lymph node examination also has been used to assess the quality of colorectal surgery 4-5. Thus, lymph node examination is of vital importance for CRC patients.

Currently, the National Comprehensive Cancer Network (NCCN) and the American Society of Clinical Oncology (ASCO) recommend that at least 12 lymph nodes should be examined. However, lymph node examination could be influenced by gender, age, tumor stage, tumor differentiation and various factors 6-9. Thus, the characteristics influencing lymph node examination should be comprehensively evaluated and taken into consideration when assessing the risk of inadequate lymph node examination.

Risk prediction model could identify individuals at high risk of developing the disease and identify new risk factors for the disease 10. Recently, several risk models for predicting incidence and prognosis of CRC have been developed and validated in different populations 10-13. Thus, risk prediction model for lymph node examination provide the opportunity to identify the risk factors regarding lymph node examination and assess the individuals' risk level of inadequate lymph node examination. However, relevant prediction model for colon cancer patients was still lacking.

Thus, the aim of the study was to develop a clinical model to predict the risk of inadequate lymph node examination before colon cancer resection and to further provide reference for surgeons and pathologists.

## Materials and Methods

### Data source and study design

In this retrospective study, we extracted colon cancer patients diagnosed between 2010 and 2014 from the Surveillance, Epidemiology, and End Results (SEER) database. The SEER database consists of 17 population-based cancer registries that represent approximately 28% of the population of the United States 14. In this study, the inclusion criteria were: 1) Patients diagnosed as stage I-III colon cancer and 2) patients underwent radical resection as the first course of treatment were included in this study. The exclusion criteria were: 1) Patients with incomplete information; 2) Patients diagnosed as rectosigmoid colon cancer or rectal cancer; 3) Patients in stage IV or did not accept radical resection as the first course of treatment; 4) Patients associated with T0/Tx, other types of histology (except for adenocarcinoma and mucinous/signet-ring cell carcinoma) and multiple primary colorectal cancer; 5) Patients underwent preoperative chemoradiotherapy were excluded from this study since the treatment would decrease the number of nodes examined. Accordingly, in the SEER database, a total of 24284 patients diagnosed as stage I-III colon cancer from 2010 to 2014 were finally enrolled in this study.

The patients extracted from the SEER database were randomly divided into two cohorts with the ratio of 1:1 using the statistical software package SPSS 22.0 (IBM Corp, Armonk, NY, USA), each cohort consisted of 12142 cases. The development cohort was used to develop the risk model while the internal validation cohort was used to validate the model. In addition, we also enrolled an external validation cohort in order to validate the model formula and risk scores in Chinese population. The inclusion criteria and exclusion criteria have been described above. Accordingly, a total of 680 patients diagnosed as stage I-III colon cancer from January 2012 to December 2014 at the Second Affiliated Hospital of Harbin Medical University were enrolled in this study. The baseline characteristics of three cohorts were shown in Table 1.

In addition, the definition of right-sided colon and left-sided colon lesion was in accord with previous study 14-15. Right-sided colon cancer was defined as location of the tumor, including the cecum, ascending colon, hepatic flexure and proximal transverse colon (proximal two-thirds of the transverse colon) while left-side colon including the distal transverse colon (distal one-third of the transverse colon), splenic flexure, descending colon and sigmoid colon.

### Candidate predictors

According to our previous study, the predictors for the model were age (categorize as < 40, 40-59, 60-79 and ≥ 80), sex (categorize as male and female), tumor size (categorize as < 5 cm and ≥ 5 cm) and tumor site (categorize as right-sided colon and left-sided colon) 6. Our previous study has shown that these patient-related predictors were associated with lymph node examination 6. Gender and age were baseline characteristics of patients while tumor site and tumor size could be determined by preoperative coloscopy. In addition, we also used preoperative carcinoembryogenic antigen (CEA) level as candidate predictor (categorize as negative and positive). In general, five patient-related factors were enrolled in this study as candidate predictors and all the predictors could be obtained before the surgical operation.

### Logistic regression model

The logistic regression model was used to predict the risk of inadequate lymph node examination. All models were built using a maximum likelihood estimation approach based on adaptive quadrature 16. This full model approach using a small number of pre-established candidate variables attempts to avoid overfitting and selection bias that is known to occur when variables are included in a risk model based on significance testing in univariate analysis 17. Each predictor in the model was associated with a score. The score was calculated by dividing the regression coefficient (β coefficient) of each predictor by the lowest β coefficient in the model, and rounding to the nearest whole number 18. The total risk score for individual was calculated by summing the score for each present risk factor. Predicted outcome rate was calculated for each patient using the model regression formula 16.

Discrimination, the ability of a predictive model to separate those who experience an event from those who do not, was assessed using the C statistic and the area under the receiver operating characteristic curve (AUC) 19. The overall C statistics and its 95% confidence intervals (CI) were calculated by logistic regression. Calibration is another measure of performance of a prediction model that tests how closely predicted outcomes agree with actual outcomes 19-20. The patients were divided into different score groups and the actual rate of inadequate lymph node examination was calculated in each group and was named as observed rate. The predicted rate of each group was calculated according to the mean predicted rate and the standard deviation (SD) 16. Finally, the predicted rate of each group was compared with the observed rate of each group to test the model calibration.

### Statistical analysis

Categorical variables were compared between groups using Chi-square test. All the statistical analyses were carried out by using the statistical software package SPSS 22.0 (IBM Corp, Armonk, NY, USA). All statistical tests were two-sided P values and a P value of < 0.05 was defined to be statistical significance.

## Results

### Univariate analysis of the candidate predictors in the development cohort

In this study, the candidate predictors for the model were age, sex, tumor size, tumor site and CEA level. In the development cohort, the rate of examining ≥ 12 lymph nodes of each subgroup was shown in Table 2. According to our results, patients in subgroup of female, right-sided colon, tumor size ≥ 5 cm, CEA positive and patients aged < 40 had higher rates of adequate lymph node examination. All the predictors except for CEA were significantly different between subgroups in the univariate analysis and they were subsequently enrolled in the logistic regression model for further analysis.

### Developing the risk prediction model

Predictors including age, sex, tumor size and tumor site were enrolled in the logistic regression model. The Hosmer-Lemeshow test (H-L test) for the model indicated no significant lack of fit (χ^2^=6.32, P=0.611). The adjusted odds ratio (95% confidence intervals, CI) between different subgroups were shown in Table 3. All the predictors were significantly associated with the risk of inadequate lymph node examination in the logistic regression analysis.

According to the results, subgroup of male, left-sided colon, tumor size < 5 cm and patients aged 40-59, 60-79 and ≥ 80 were risk factors for inadequate lymph node examination in logistic regression analysis. The β coefficient of each risk factor was calculated by regression formula and risk score for each factor was calculated according to their β coefficient. The risk score of each predictor was also shown in Table 3.

The total risk score of each patient in the development cohort was calculated by summing the scores of each present risk factor. The highest total risk score was 15 and the lowest was 0. In addition, the predicted rate of each patient was also calculated according to the model formula and was attached to each patient in the development cohort.

### Outcomes of internal and external validation cohort

We also performed univariate analysis in the internal validation cohort and external validation cohort. Since the CEA had no significant association with risk of inadequate lymph node examination in the development cohort, other predictors were used as candidate predictors for further analysis in two validation cohorts. According to our results, all of the four candidate predictors were significantly associated with lymph node examination in the univariate analysis in two validation cohorts. The characteristics and results of univariate analysis of these two cohorts were shown in Table 4 and Table 5.

We also performed logistic regression analysis in the internal validation cohort and external validation cohort to calculate the predicted rate, the significance of each predictor and to validate the feasibility of the model. All the predictors in the internal validation cohort and external validation cohort were significantly associated with risk of inadequate lymph node examination (data not shown). The highest total risk score of both validation cohort was 15 and the lowest total risk score was 0. The H-L test for internal validation cohort (χ^2^=10.73, P=0.218) and external validation cohort (χ^2^=10.81, P=0.147) showed that the model fitted well for these two validation cohorts.

### Model discrimination and calibration

**Figure 1:**
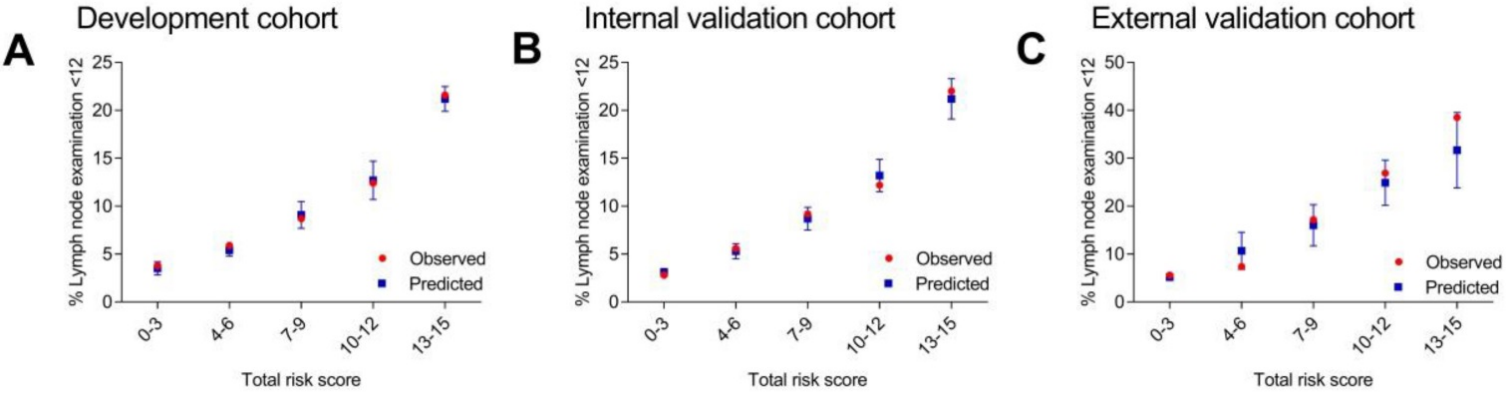
Observed and predicted rates of inadequate lymph node examination according to different score groups.

C statistics were carried out in all the three cohorts. The results of AUC were 0.66 (95% CI, 0.63-0.67) for the development cohort, 0.66 (95% CI, 0.64-0.68) for the internal validation cohort and 0.73 (95% CI, 0.68-0.78) for the external validation cohort. Then, the patients were divided into different score groups according to total risk scores for model calibration. The score groups were divided into 0-3, 4-6, 7-9, 10-12 and 13-15. The mean predicted rate, SD and the observed rate were calculated for different score groups. The results of observed rate and predicted rate of inadequate lymph node examination (< 12 nodes) for each score group were shown in Figure 1 and Supplementary Table 1. Generally, in each score group, the observed rate was in accord with the predicted rate which represented that the model calibration was well. These results indicated that the scoring system were feasible for the patients in the SEER database (American population) and the patients in the Second Affiliated Hospital of Harbin Medical University (Chinese population).

### Category of risk group

The risk level of each patient was classified as low, medium and high risk. Patients scored 0-6 were divided into low risk group (highest predicted rate of 5.4%) while patients scored ≥ 13 were divided into high risk group (lowest predicted rate of 21.2%) according to their result in development cohort. Other patients scored 7-12 were divided into medium risk group. In other two validation cohorts, we also found that the lowest predicted rate of high risk group (scored ≥ 13) was three times more than the highest predicted rate of low risk group (scored 0-6). Thus, the category of low, medium and high risk group was in accord with total risk score of 0-6, 7-12 and ≥ 13.

## Discussion

Lymph node examination plays an important role in evaluating the quality of surgery and pathological examination which associated with accurate staging and performance of adjuvant treatment 14. However, it can be influenced by various factors, including the extent of surgical resection, the technique of pathology examination and the skill of surgeons and pathologists 16,21. In addition, patient-related factors also influenced the number of nodes examined. Our previous study had shown that predictors including T stage, N stage, age, sex, grade, histology, tumor size and tumor location were associated with the median number of lymph node, the rate of ≥ 12 lymph nodes and the rate of node positive of colon cancer patients 6. Current studies regarding lymph node examination mostly focused on assessing the risk factors associated with lymph node examination while ignored assessing the weight of each factor and the total risk level of each patient.

Here, we extracted patients from American population and Chinese population in order to develop a risk prediction model and validate if the model apply to different populations. The predictors were chosen according to our previous study and the category of these predictors were the same with our previous study 6. In addition, CEA level before surgery was also used as a predictor in this study since it was an important result of preoperative examination. We firstly carried out logistic regression analysis in development cohort. According to model formula, the risk score of each predictor was calculated. The different score of each predictor indicated the different weight of each predictor and they could influence the lymph node examination to what extent. For each score group, the mean predicted rate and its standard deviation (SD) could form a predicted rate interval. The observed rate of each score group should be included in the predicted rate interval of the same score group which represent the model calibration was well. Generally, the outcome of development cohort indicated that the model was well established. This full model approach have been tested by various studies regarding risk prediction model and have been proved to be efficient 17-20.

In addition, univariate analysis and logistic regression analysis were also performed in the internal validation cohort and external validation cohort. The risk scores have been determined by the development cohort while the predicted rate would be recalculated according to different model formula of two validation cohorts. The results showed that the model was well validated in these two cohorts. Thus, the model discrimination and calibration was successfully performed in all cohorts. Finally, the category of low, medium and high risk was corresponding to total risk score group 0-6, 7-12 and 13-15. The internal validation cohort was used to validate the model parameter and the risk score which indicated the scoring system and the category of risk level was apply to SEER database (American population). The external validation cohort was used to validate the scoring system and the category of risk level to prove that these were also applied to Chinese population.

According to our results, the patients in high risk group might expose to inadequate lymph node examination, surgeons and pathologists needed to pay close attention to these patients. Compared with previous studies, the model could provide useful reference for surgeons and pathologists before the surgical procedures and pathological examinations were carried out. However, these conclusions only provide potential reference but not guidance. The medical decision would finally be made according to the reference of the risk prediction model, surgeons and pathologists' skill and the actual condition of the patients.

Since lymph node examination is of vital importance for colon cancer patients, relevant studies have been performed globally. Here, we would like to suggest that we had novel approaches in two aspects. For one thing, compared with previous studies, we performed univariate analysis and logistic regression model to identify risk factors and we further used the model formula to develop a scoring system and risk level category which could be helpful to easily recognize individual's risk level of inadequate lymph node examination before surgery. For another, we validated the model in two cohorts and found the model applied to American population and Chinese population. Thus, our research has specific novel approaches.

The researchers acknowledged several limitations of this study. In this retrospective study, the selection basis could not be avoided. In addition, only patient-related characteristics were used to build the risk prediction model. Several hospital-related factors could influence the lymph node examination either but they were not enrolled in this study. At last, the factors included in this study were limited and the lack of novel risk factors would also be the limitation of this study. Despite these limitations, this study developed and validated a preoperative risk prediction model for inadequate lymph node examination of colon cancer patients for the first time and put forward a scoring system and risk level category to calculate the individualized rate of inadequate lymph node examination. In addition, we also used an external validation cohort to validate whether the prediction model was applied to both American population and Chinese population which would be an advantage of our study. Our research could give reference to surgeons and pathologists which might be helpful to individualized medical decision.

## Supplementary Material

Supplementary table.

## Figures and Tables

**Table 1:** Baseline characteristics of Development cohort, Internal validation cohort and External validation cohort.

Characteristics		Development cohort (n,%)	Internal validation cohort (n,%)	External validation cohort (n,%)	*P value*
**Gender**	Female	6127 (50.4)	6214 (51.2)	284 (41.8)	P<0.001
	Male	6015 (49.6)	5928 (48.8)	396 (58.2)	
**Age (years)**	< 40	317 (2.6)	304 (2.5)	26 (3.8)	P<0.001
	40-59	3011 (24.8)	2946 (24.3)	238 (35.0)	
	60-79	6026 (49.6)	6044 (49.8)	386 (56.8)	
	≥ 80	2788 (23.0)	2848 (23.4)	30 (4.4)	
**Tumor site**	Right-sided colon	7839 (64.6)	7760 (63.9)	347 (51.0)	P<0.001
	Left-sided colon	4303 (35.4)	4382 (36.1)	333 (49.0)	
**Tumor size (cm)**	< 5	7032 (57.9)	7002 (57.7)	393 (57.8)	P=0.927
	≥ 5	5110 (42.1)	5140 (42.3)	287 (42.2)	
**Node examined**	≥ 12	10822 (89.1)	10818 (89.1)	561 (82.5)	P<0.001
	< 12	1320 (10.9)	1324 (10.9)	119 (17.5)	
**CEA**	Negative	8080 (66.5)	7976 (65.7)	434 (63.8)	P=0.208
	Positive	4062 (33.5)	4166 (34.3)	246 (26.2)	

**Table 2:** Univariate analysis of candidate predictors in the Development cohort.

Characteristics		Number	No. of examine ≥12 nodes	*P value*
**Gender**	Female	6127	5518 (90.1)	P=0.001
	Male	6015	5304 (88.2)	
**Age (years)**	< 40	317	301 (95.0)	P<0.001
	40-59	3011	2716 (90.2)	
	60-79	6026	5311 (88.1)	
	≥ 80	2788	2494 (89.5)	
**Tumor site**	Right-sided colon	7839	7202 (91.9)	P<0.001
	Left-sided colon	4303	3620 (84.1)	
**Tumor size (cm)**	< 5	7032	6061 (86.2)	P<0.001
	≥ 5	5110	4761 (93.2)	
**CEA**	Negative	8080	7179 (88.8)	P=0.086
	Positive	4062	3643 (89.7)	

**Table 3:** Results of logistic regression analysis and risk scores of the Development cohort.

Characteristics		Adjusted OR (95% CI)	*P value*	*β coefficient*	Score
**Gender**	Female	1.0 (ref)	P=0.009	-	0
	Male	1.2 (1.0-1.3)		0.16	1
**Age (years)**	< 40	1.0 (ref)	P<0.001	-	0
	40-59	1.8 (1.1-3.1)		0.60	3
	60-79	2.5 (1.5-4.2)		0.92	5
	≥ 80	2.6 (1.6-4.4)		0.96	6
**Tumor site**	Right-sided colon	1.0 (ref)	P<0.001	-	0
	Left-sided colon	2.1 (1.9-2.4)		0.76	4
**Tumor size (cm)**	≥ 5	1.0 (ref)	P<0.001	-	0
	< 5	2.0 (1.8-2.3)		0.70	4

**Table 4:** Univariate analysis of candidate predictors in the Internal validation cohort.

Characteristics		Number	No. of examine ≥12 nodes	*P value*
**Gender**	Female	6214	5595 (90.0)	P=0.001
	Male	5928	5223 (88.1)	
**Age (years)**	< 40	304	287 (94.4)	P<0.001
	40-59	2946	2677 (90.9)	
	60-79	6044	5366 (88.8)	
	≥ 80	2848	2488 (87.4)	
**Tumor site**	Right-sided colon	7760	7105 (92.2)	P<0.001
	Left-sided colon	4382	3713 (84.7)	
**Tumor size (cm)**	< 5	7002	6017 (85.9)	P<0.001
	≥ 5	5140	4801 (93.4)	

**Table 5:** Univariate analysis of candidate predictors in the External validation cohort.

Characteristics		Number	No. of examine ≥12 nodes	*P value*
**Gender**	Female	284	249 (87.7)	P=0.002
	Male	396	312 (78.8)	
**Age (years)**	< 40	26	23 (88.5)	P<0.001
	40-59	238	199 (87.3)	
	60-79	386	322 (83.4)	
	≥ 80	30	17 (56.7)	
**Tumor site**	Right-sided colon	347	319 (91.9)	P<0.001
	Left-sided colon	333	242 (72.7)	
**Tumor size (cm)**	< 5	393	307 (78.1)	P<0.001
	≥ 5	287	254 (88.5)	

## Abbreviations

CC: colon cancer
CRC: colorectal cancer
NCCN: National Comprehensive Cancer Network
ASCO: American Society of Clinical Oncology
SEER: Surveillance, Epidemiology and End Results
CEA: carcinoembryogenic antigen
NY: New York
USA: United States of America
AUC: area under the receiver operating characteristic curve
CI: confidence intervals
SD: standard deviation

## References

[1] Siegel RL, Miller KD, Jemal A (2019). Cancer statistics, 2019. CA Cancer J Clin.

[2] Norwood MG, Sutton AJ, West K (2010). Lymph node retrieval in colorectal cancer resection specimens: national standards are achievable, and low numbers are associated with reduced survival. Colorectal Dis.

[3] Swanson RS, Compton CC, Stewart AK (2003). The prognosis of T3N0 colon cancer is dependent on the number of lymph nodes examined. Ann Surg Oncol.

[4] Manwaring ML, Ko CY, Fleshman JW Jr (2012). Identification of consensus-based quality end points for colorectal surgery. Dis Colon Rectum.

[5] Dimofte G, Târcoveanu E, Taraşi M (2011). Mean number of lymph nodes in colonic cancer specimen: possible quality control index for surgical performance. Chirurgia (Bucur).

[6] Guan X, Chen W, Li S (2016). Alterations of lymph nodes evaluation after colon cancer resection: patient and tumor heterogeneity should be taken into consideration. Oncotarget.

[7] Nedrebø BS, Søreide K, Nesbakken A (2013). Risk factors associated with poor lymph node harvest after colon cancer surgery in a national cohort. Coloretal Dis.

[8] Khan H, Olszewski AJ, Somasundar P (2014). Lymph node involvement in colon cancer patients decreases with age; a population based analysis. Eur J Surg Oncol.

[9] Tekkis PP, Smith JJ, Heriot AG (2006). A national study on lymph node retrieval in resectional surgery for colorectal cancer. Dis Colon rectum.

[10] Win AK, Macinnis RJ, Hopper JL (2012). Risk prediction models for colorectal cancer: a review. Cancer Epidemiol Biomarkers Prev.

[11] Ma E, Sasazuki S, Iwasaki M (2010). 10-Year risk of colorectal cancer: development and validation of a prediction model in middle-aged Japanese men. Cancer Epidemiol.

[12] Fieber JH, Sharoky CE, Collier KT (2018). A preoperative prediction model for risk of multiple admissions after colon cancer surgery. J Surg Res.

[13] Shin A, Joo J, Yang HR (2014). Risk prediction model for colorectal cancer: National Health Insurance Corporation study, Korea. PLoS One.

[14] Guan X, Wang Y, Hu H (2018). Reconsideration of the optimal minimum lymph node count for young colon cancer patients: a population-based study. BMC Cancer.

[15] Imperial R, Ahmed Z, Toor OM (2018). Comparative proteogenomic analysis of right-sided colon cancer, left-sided colon cancer and rectal cancer reveals distinct mutational profiles. Mol Cancer.

[16] Gelos M, Gelhaus J, Mehnert P (2008). Factors influencing lymph node harvest in colorectal surgery. Int J Colorectal Dis.

[17] Jorgensen ML, Young JM, Dobbins TA (2015). A mortality risk prediction model for older adults with lymph node-positive colon cancer. Eur J Cancer Care (Engl).

[18] Royston P, Moons KG, Altman DG (2009). Prognosis and prognostic research: developing a prognostic model. BMJ.

[19] Lee SJ, Lindquist K, Segal MR (2006). Development and validation of a prognostic index for 4-year mortality in older adults. JAMA.

[20] Liu J, Hong Y, D'Agostino RB Sr (2004). Predictive value for the Chinese population of the Framingham CHD risk assessment tool compared with the Chinese Multi-Provincial Cohort Study. JAMA.

[21] Guan X, Chen W, Jiang Z (2016). Exploration of the optimal minimum lymph node count after Colon Cancer resection for patients aged 80 years and older. Sci Rep.

