# Supplementary material for: A Preoperative Risk Prediction Model for Lymph Node Examination of Stage I-III Colon Cancer Patients: A Population-Based Study
Source: J Cancer. 2020 Mar 5;11(11):3303–9. doi: 10.7150/jca.41056 (PMC7097944; doi:10.7150/jca.41056)
Supplement: Supplementary file 1 — Supplementary table. [file jcav11p3303s1.pdf]

Supplementary Table 1 Observed and predicted rates of examined < 12 nodes according to different scoring groups.

	Development cohort		Internal validation cohort		External validation cohort	
Risk score	Observed (%)	Predicted (%)	Observed (%)	Predicted (%)	Observed (%)	Predicted (%)
0-3	3.8	3.5	2.8	3.1	5.6	5.2
4-6	5.9	5.4	5.6	5.3	7.4	10.7
7-9	8.7	9.1	9.2	8.7	17.2	16.0
10-12	12.4	12.7	12.2	13.2	26.9	24.9
13-15	21.6	21.2	22.0	21.2	38.5	31.7
